# To determine the effect of long-term antiepileptic drug on the serum folate and vitamin B12 among epileptic patients

**DOI:** 10.1038/s41598-021-83312-y

**Published:** 2021-02-23

**Authors:** M. Kathiravan, S. Kavitha, R. Shanthi

**Affiliations:** 1 Department of Information Technology, Rajalakshmi Engineering College, Chennai, 602105 India; 2 Department of Bio Chemistry, Government Chengalpattu Medical College, Chennai, 603001 India; 3 Department of Bio Chemistry, Government Omandurar Medical College, Chennai, 600002 India

**Keywords:** Biochemistry, Biological techniques, Drug discovery, Medical research

## Abstract

The purpose of this study was to prevent anaemia caused on long-term phenytoin treatment among Epileptic patients. Therefore, two groups were categorised based on the duration of phenytoin namely, cases and control. The duration of Phenytoin treatment for case was > 2 years while control had < 1 year. The estimation of serum Folate and Vitamin B12 was carried out and the association between the deficiency of Vitamins with the duration of treatment were evaluated using Linear Regression Analysis. The statistical results shown that the mean value of Folate (2.61 ± 1.81) and the mean value of Vitamin B12 (284.68 ± 110.12) were considered to be low as the duration of Phenytoin treatment increases (> 2 years) compare to control with the Folate concentration of (7.01 ± 4.40) which was statistically significant (p < 0.000). The experimental results have proved that a long-term phenytoin treatment significantly affects the concentration of Folate and Vitamin B12 among the Epileptic patients vigorously.

## Introduction

Epilepsy is a chronic neurological disorder caused by transient cerebral dysfunction of human brain. Many factors lead to the cause of this disorders and infections like meningitis or encephalitis. The prevalence rate of this disease in India is 5.59/1000 population with no statistical rate of differences between men and women^1^, while the worldwide prevalence is (7.0%)^2^.

The widely practised antiepileptic drug for this disease is Phenytoin. The long-term treatment of this drug develops a deficiency of Folate and Vitamin B12 which in turn promotes anaemia to a significant degree^3^. The former studies also had shown that the bioavailability and metabolism of Folate and Vitamin B12 were altered while the duration of Phenytoin treatment was increased^4^. The manifestation of these two deficiencies are megaloblastic anaemia, cognitive impairment and depression.

Despite the use of newer antiepileptic drugs like Topiramate and Lamotrigine, Phenytoin remains most preferred since it has a broad spectrum of activity and tolerability among Epileptic patients^5^. Though we had a various treatment plan based on the severity of the symptoms, yet the treatment with medications control Epilepsy only to an extended level rather than a complete cure. Hence, the need of supplementing Vitamin B12 and Folate are required to be determined depending on the duration of Phenytoin treatment.

The serum concentration of Vitamin B12 and Folate were reduced significantly after the treatment^2^. Hence the proposed study was focussed Epileptic patients who were on Phenytoin treatment. And, while a recent work was reported with large samples to find the prostate cancer risk of patients who consume metformin drugs for antidiabetic treatment^6^, Differently, this study was focussed to prevent anaemia caused on long-term phenytoin treatment among Epileptic patients.

A recent survey conducted by the Neurology department at Stanley Medical College & Hospital (SMCH) indicated a significant increase in the number of Epileptic patients on Phenytoin Monotherapy during the period of 2015 to 2018. According to the statistics of our tertiary Hospital, over a half of patients on long-term Phenytoin therapy have experienced a low concentration of Vitamin B12 and Folate. Regarding this a serious investigation was conducted by SMCH and confirmed the deficiency of both Vitamins caused for megaloblastic anaemia on Epileptic patients. Hence, this motivates the core concern of study and research.

## Methods and materials

### Ethics, consent and permission

The Department of Neurology, Department of Biochemistry of SMCH had been the major centres for the study. The request for an approval from the Institutional Ethical Committee (IEC) was considered at the IEC meeting held on 21.12.2017 in the Council Hall, SMCH, Chennai at 10 AM. The Principal investigator and their team are directed to adhere to the guidelines. They are, changes in the study procedure, site investigator investigation and guide must be approved by IEC. The area of the work that are applied for ethical clearance should not be deviated. Any adverse or serious adverse reaction must be brought into IEC. The work must be completed within the specified period of time. Finally, the summary of the work should be submitted to IEC on completion of the work. The members of the committee, the secretary and the chairman were pleased to approve the proposed work submitted by the principal investigator and the same has been approved in accordance with the guidelines and regulations of IEC in SMCH. Also, a written consent was obtained from each participant after providing a complete explanation of the study prior to examination.

### Subject requirements

The design for this analysis was chosen to be cross sectional study. The study was conducted in a tertiary care Hospital during the course of 6 months which was between January 2018 to June 2018. The research was performed over 100 Epileptic patients on Phenytoin monotherapy excluding certain criteria such as age below 18 years; pregnancy; heart, liver and kidney dysfunction; drugs namely, oral contraceptives, Pantoprazole and Methotrexate; alcohol and smoking.

### Definition of disease

Estimate and analyse the deficiencies of Vitamin B12 and Folate among Epileptic patients on long-term Phenytoin treatment. The number of participants chosen for each category were 50. While the control group comprises newly diagnosed Epileptic patients which was especially < 1 year, cases include > 2 years. Different age groups were compared for both cases and control to calculate Vitamins deficiencies. The age distribution for both cases and control were classified as 18–25 years, 26–50 years and above 50 years. A p value of < 0.05 was considered to be statistically significant.

### Estimations

The procedure started with the examination of blood samples collected from the participants under strict aseptic precautions. Around 5 ml of blood was obtained from each person in which 3 ml taken in plain red topped vein puncture tubes without any additives or gel barrier. Remaining 2 ml was taken in EDTA tubes to analyse the complete blood count and peripheral smear examinations^8^. The collected samples were centrifuged at 2000–2500 rpm for 10 min followed by an immediate separation of serum, which was stored at minus 20 °C in a deep freezer for the estimation of Vitamin B12 and Folate. Examining the participants, various investigations such as Glucose, Urea and Creatinine were performed. Aperture-Impedance Method^7^ was applied to determine the count of Haemoglobin, total WBC, and differential, RBC, Platelet, MCV, MCH and MCHC.

### Estimation of serum vitamin B12

The estimation of serum Vitamin B12 was performed with cobas e 411/601 analyzer. The reagents working solutions include the rack pack, which is the kit placed on instrument; Streptavidin coated microparticles; Ruthenium labelled intrinsic factor acting as reagent 1; Vitamin B12 labelled biotin acting as reagent 2; Dithiothreitol as pre-treatment 1 and Sodium hydroxide or sodium cyanide as pre-treatment 2. Quantitative determinations of Vitamin B12 and Folate were performed using Electro-chemiluminescence immunoassay method. Immunoassay analysers based on electro chemiluminescent technology used Ruthenium complex and the measuring cell. “Electro” means electrical stimulation, “chemi” refers to chemical reaction and luminescence indicates production of light. Total duration of the assay was figured to be 27 min. In the first incubation, 15 µl of sample was incubated with pre-treatment 1 and 2 for 9 min so as to release the bound Vitamin B12. The second incubation was also performed for 9 min in which the above pre-treated sample was incubated with Ruthenium labelled intrinsic factor to form Vitamin B12 binding protein complex. The amount of this complex depends on the analyte level of the sample. In the final incubation, which was also performed for 9 min, the above complex reacts with streptavidin coated microparticles and Vitamin B12 labelled with biotin to form ruthenium labelled intrinsic factor Vitamin B12 biotin complex. The entire complex is bound to the solid phase via interaction of biotin and streptavidin. The reagent was Vitamin B12 Elecsys from Roche diagnostics. In regard to storage and stability, it was stored under 2–8 °C and not to be freezed. The reference range for the Vitamin B12 was 200–700 pg/ml.

### Estimation of folate

The estimation of Folate was performed for a total of 27 min. The reagent was Folate reagent from Roche diagnostics. In the first incubation, 25 µl of sample was incubated with pre-treatment 1 and 2 for 9 min, so as to release the bound Folate. The second incubation was also performed for 9 min in which the above pre-treated sample was incubated with ruthenium labelled intrinsic factor to form Folate binding protein complex. The amount of this complex depends on the analyte level of the sample. In the final incubation, which was also performed for 9 min, the above complex reacts with streptavidin coated microparticles and Vitamin B12 labelled with biotin to form ruthenium labelled intrinsic factor Folate biotin complex. The entire complex is bound to the solid phase via interaction of biotin and streptavidin. The reaction mixture was aspirated into the measuring cell where the micro-particles were magnetically captured onto the surface of the electrode. Then, the unbound substances were removed. The application of a voltage to the electrode then induces chemiluminescent emission, which was measured by a photomultiplier. The results were determined with the help of a calibration curve generated by 2-point calibration. In regard to storage and stability, it was stored under 2–8 °C. If unopened, it was stored at 2–8 °C up to the expiry date. After opening the storage was 2–8 °C for 12 weeks. If on an analyser, it can be stored for 8 weeks. The reference range for the Folate was 4–20 ng/ml. Calibrations were performed once per reagent lot using fresh reagent. Two levels of calibrators CAL 1 and CAL 2 were provided in separate kits for each analyte.

### Statistical analysis and results

The results obtained for cases were compared with controls by student's unpaired ‘t’ test. Pearson’s correlation coefficient was used to estimate the degree of association between two quantitative variables. The mean value of age was 38.12 for cases and 39.66 for control and the standard deviation was 12.9 and 14.7 respectively. No statistical significance (P = 0.36) was obtained with regard to age distribution between cases and control. Hence, both the groups were comparable.

The significance of Vitamin B12 and Folate concentration for both the groups were shown (Table 1a). While the mean value of Vitamin B12 was 284.68 for cases, the control was 469.44. While the standard deviation for case was 110.12, the control values were 154.32. Thus, shows the significant difference between the mean values of Vitamin B12 for cases and control (p = 0.01). Meanwhile, the mean value of Folate for cases were 2.61 and control was 7.01. The standard deviation was 1.81 and 4.40 for cases and control respectively. This finding shows a significant difference between the mean values of Folate for cases and control (p < 0.000).

**Table 1:** Statistical analysis.

Group	Count	Vitamin B12	P value	Folate	P value
**1a Significance of vitamin B12 and folate concentration**					
Cases	50	284.68 ± 110.12	0.01	2.61 ± 1.81	< 0.000
Control	50	469.44 ± 154.32		7.01 ± 4.40	
**1b Year wise distribution of vitamin B12 and folate concentration depending on the duration of cases**					
2–5	14	315.25 ± 114.76	0.23	3.86 ± 2.95	0.004
5–10	29	283.93 ± 112.52		2.27 ± 0.65	
> 10	7	227.99 ± 74.04		1.5 ± 0.36	

Variables	Pearson’s correlation coefficient (r)	Significance (p)	Interpretation		
**1c Correlation of vitamin B12 and folate concentration with duration of cases**					
Vitamin B12 versus duration	− 0.09	0.02	Significant and negative correlation		
Folate versus duration	− 0.09	0.001	Significant and negative correlation		
**1d Correlation of vitamin B12 and folate concentration with duration of control**					
Vitamin B12 versus duration	0.00	0.78	Not significant and no correlation		
Folate versus duration	0.00	0.66	Not significant and no correlation		

The different group of patients for both Vitamin B12 and Folate concentration was shown (Table 1b). The patients with Phenytoin treatment for 2–5 years were 14, having a mean of 315.25 and 114.76 as standard deviation. The next group holds 29 patients for 5–10 years has a mean value of 283.93 and 112.52 as the standard deviation. The last group > 10 consists 7 patients with the mean as 227.99 and standard deviation was 74.04 with a P value of 0.23. The Folate count of patients was 14 for 2–5 years with a mean of 3.86 along with a standard deviation 2.95. The next group consists of 29 with the mean of 2.27 and 0.65 as the standard deviation. The last group has 7 patients with the mean as 1.5 and 0.36 as the standard deviation along with P value 0. 004. These findings showed a decrease in the mean of Vitamin B12 and Folate concentration according to the duration of Phenytoin treatment. While the Vitamin B12 (0.02) and Folate (0.00) values have significant and negative correlation with the duration of Phenytoin treatment for cases (Table 1c), the Vitamin B12 (0.78) and Folate (0.66) values for control were not significant and have no correlation with the duration of Phenytoin treatment (Table 1d).

### Linear regression

**Figure 1:**
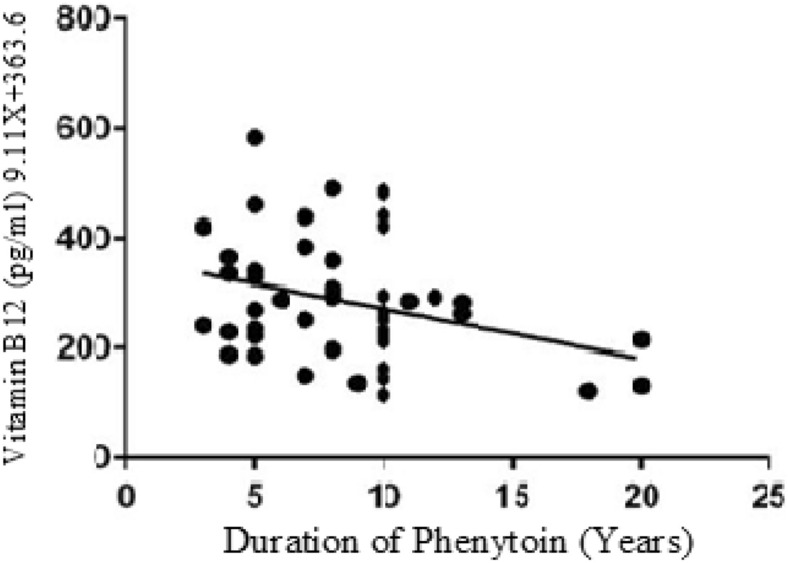
Analysis between duration of phenytoin treatment and vitamin B12 for cases.

**Figure 2:**
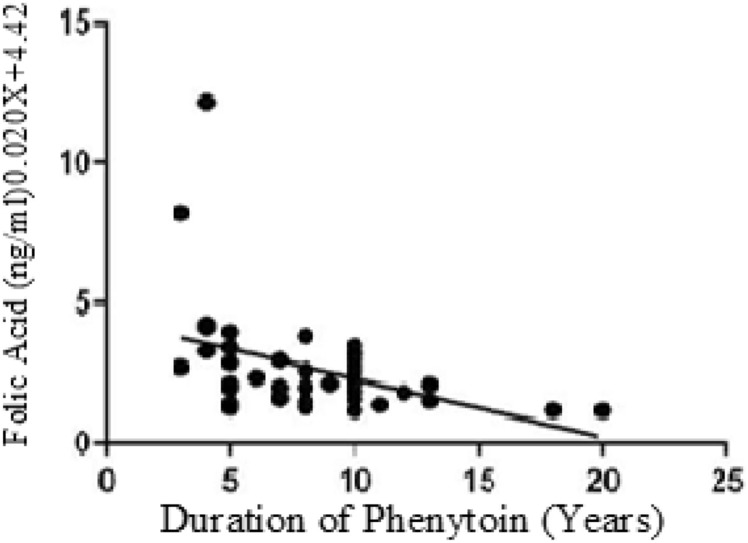
Analysis between duration of phenytoin treatment and folate for case.

**Figure 3:**
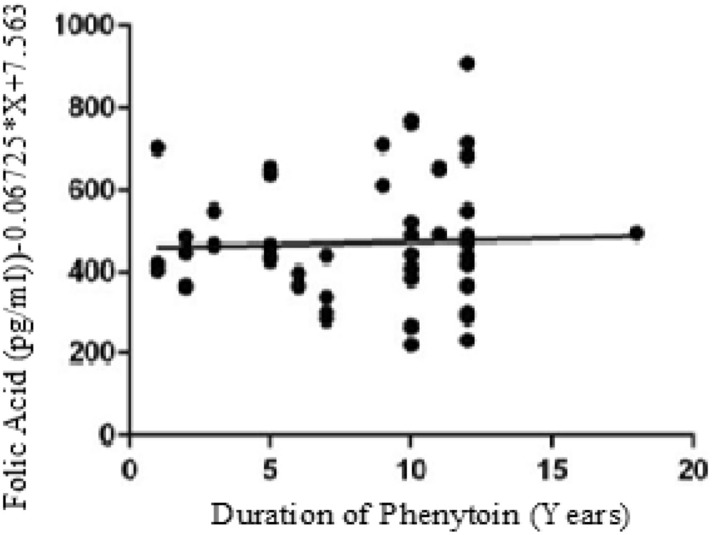
Analysis between duration of phenytoin treatment and vitamin B12 for control.

**Figure 4:**
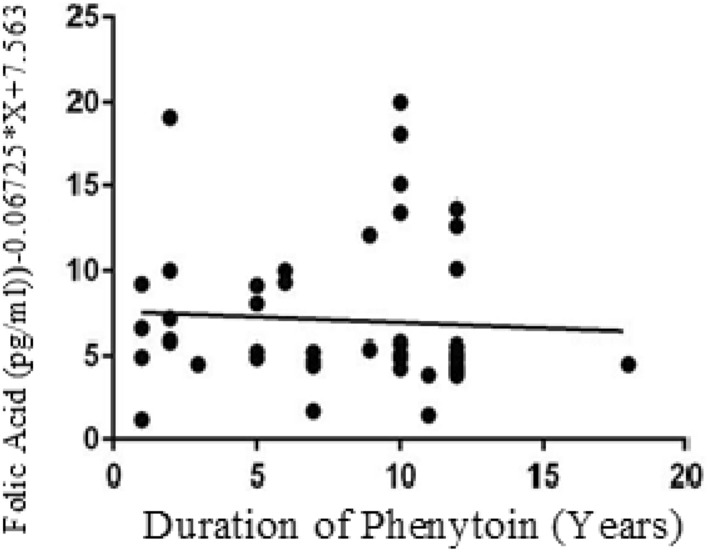
Analysis between duration of phenytoin treatment and folate for control.

The below figures have divulged the correlation of Vitamins with duration of Phenytoin treatment. The Vitamin B12 values have negative correlation as linear regression analysis has downward slope with r value of − 0.09 (Fig. 1). Similarly, the Folate values have negative correlation since the graph has downward slope with r value − 0.19 in cases (Fig. 2). While cases have downward slope, the Vitamin B12 and Folate for control have no correlation with duration of Phenytoin with r value as 0.00 (Fig. 3) and 0.00 (Fig. 4).

### Ethics approval and consent to participate

The research was performed on 100 Epileptic patients on Phenytoin monotherapy excluding certain criteria such as age below 18 years; pregnancy; heart, liver and kidney dysfunction; drugs namely, oral contraceptives, Pantoprazole and Methotrexate; alcohol and smoking. The request for an approval from the Institutional Ethical Committee was considered on the meeting held on 21.12.2017, Stanley Medical College, Chennai. Written informed consent was obtained from all enrolled patients and healthy individuals.

## Discussions

The proposed study has many interesting measures compared to previous researches. The mean value of serum B12 concentration has a statistically significant fall of 39.4% compared to the mean value of the control group (Table 1a). This finding is consistent with a result of Linnebank et al. 2011^15^, Sener et al. 2006^12^ and Majola et al. 2000^13^. The decrease of Vitamin B12 concentration was due to the effect of Phenytoin on bioavailability and metabolism of Vitamin B12^9,10^. The mean value of Vitamin B12 (Table 1b) for various categories was consistent with the result of Linnebank et al. 2011^15^ and Sener et al. 2006^12^. Similarly, the mean value of serum Folate concentration had shown a statistically significant fall of 62.8% compared to the control group. The decrease of Folate concentration was due to the effect of Phenytoin mainly on inhibiting the intestinal absorption of Folate 1. The result showed that the mean value of Folate concentration has decreased, while the duration of Phenytoin treatment was increased for cases compared to control. That is, the mean value of Folate (Table 1b) for the above said categories were statistically significant. Thus, the above result was consistent with the finding of Kishi et al. 1997^11^, Davis et al. 2002^14^ and Abhishek Singh et al. 2013^16^.

The analysis also had some interesting findings compared to other research. While 11 patients had a Vitamin B12 deficiency (< 200 pg/ml) and 18 with Folate deficiency (< 2 ng/ml), 11patients had both Vitamin B12 and Folate deficiency from the total of 50 participants in cases. That is, while 3 patients had Folate deficiency, no such issues were found for Vitamin B12 in control. Thereby, the prevalence of Folate and Vitamin B12 deficiency among cases were 36% and 22% while the control rate was 6%. These results showed that the Vitamin deficiency was increased among cases compared to control. The finding was consistent with the study of Rivey et al. 1984^17^, Tekin et al. 2016^18^ and Sampson et al. 2019^19^ which states that Folate deficiency resulting from long term Phenytoin therapy was a common occurrence but the progression of deficiency to megaloblastic anaemia was rare.

The current study has discovered a statistically significant negative correlation between Vitamin B12 concentration with the duration of Phenytoin (r = − 0.09, p = 0.02) among cases (Table 1c). This study also revealed a statistically significant negative correlation between Folate concentration with the duration of Phenytoin treatment (r = − 0.19, p = 0.001) among cases (Table 1c). The result had shown the decreased Folate concentration while the duration of Phenytoin treatment was increased.

The analysis on above study has also discovered new measures. That is, while we had a male and female ratio as 35 and 15 for cases, 28 male and 12 female patients have found deficiencies that included both concentrations. Likewise, 1 male and 2 female have got low deficiency of Folate from the total of 30 male and 20 female in control. Thereby no evidence proved that gender has impact on phenytoin monotherapy. Meanwhile, age has some impact on phenytoin treatment. That is, while 40 patients have a deficiency of phenytoin treatment, 25 of them ages were > 50 in cases. Similarly, the patients who had a mild deficiency in control were found in the same age group. Hence, this study ensures that deficiency count of Vitamin B12 and Folate was higher for aged people than other participants. This study has also addressed the seriousness of long-term Phenytoin treatment. While 14 patients had a treatment for 2–5 years, 8 were found with deficiency of Vitamins. Likewise, while 29 patients had a treatment between > 5 year and < 10 year, 25 patients were found with deficiencies and all 7 have been found for the last category. Therefore, the above analysis has witnessed that the long-term phenytoin treatment was the cause for the deficiency of Vitamin B12 or Folate concentration of Epileptic patients.

## Conclusions

The present study concluded that the Folate and Vitamin B12 deficiency was directly proportional to the duration of Phenytoin treatment. A long-term treatment was significantly affecting the Folate and Vitamin B12 concentrations among Epileptic patients vigorously. However, the patients were on Phenytoin treatment for < 1 year found with mild deficiency of Folate. Hence, the supplements of Vitamin B12 and Folate have been recommended at the beginning of Phenytoin treatment to avoid the deficiency which in turn leads to anaemia. The estimation of Methyl Malonic acid, Homocysteine, and Holotranscobolamin concentration which are metabolites of Vitamin B12 can be used to confirm diagnosis of Vitamin B12 and Folate deficiency. With a large sample size, we will focus this matter in future work.
